# Disengagement of Visual Attention in Infancy is Associated with Emerging Autism in Toddlerhood

**DOI:** 10.1016/j.biopsych.2012.11.030

**Published:** 2013-08-01

**Authors:** Mayada Elsabbagh, Janice Fernandes, Sara Jane Webb, Geraldine Dawson, Tony Charman, Mark H. Johnson, The British Autism Study of Infant Siblings Team

**Affiliations:** 1 Department of Psychiatry (ME), McGill University, Quebec, Canada; 2 Centre for Brain and Cognitive Development (ME, JF, MHJ), Birkbeck, University of London, London, United Kingdom; 3 Department of Psychiatry and Behavioral Sciences (SJW), University of Washington, Seattle, Washington; 4 Department of Psychiatry (GD), University of North Carolina at Chapel Hill, Chapel Hill, North Carolina, and Autism Speaks, New York, New York; 5 Department of Psychology, Institute of Psychiatry (TC), King's College London, London, United Kingdom

**Keywords:** Autism, disengagement, familial risk, infant, prospective study, visual attention

## Abstract

**Background:**

Early emerging characteristics of visual orienting have been associated with a wide range of typical and atypical developmental outcomes. In the current study, we examined the development of visual disengagement in infants at risk for autism.

**Methods:**

We measured the efficiency of disengaging from a central visual stimulus to orient to a peripheral one in a cohort of 104 infants with and without familial risk for autism by virtue of having an older sibling with autism.

**Results:**

At 7 months of age, disengagement was not robustly associated with later diagnostic outcomes. However, by 14 months, longer latencies to disengage in the subset of the risk group later diagnosed with autism was observed relative to other infants at risk and the low-risk control group. Moreover, between 7 months and 14 months, infants who were later diagnosed with autism at 36 months showed no consistent increases in the speed and flexibility of visual orienting. However, the latter developmental effect also characterized those infants who exhibited some form of developmental concerns (but not meeting criteria for autism) at 36 months.

**Conclusions:**

Infants who develop autism or other developmental concerns show atypicality in the development of visual attention skills from the first year of life.

Autism spectrum disorders (ASD) (henceforth autism) are primarily defined on the basis of social and communication impairment in childhood. Recent lessons from genetic studies have highlighted substantial overlap between autism and related conditions (1). Strictly defined, clinical categories do not capture our current understanding of the increasingly multidimensional and complex clinical, cognitive, and behavioral phenotypes associated with the condition (2). There is increasing interest in understanding neurodevelopmental pathways, potentially overlapping across multiple clinically defined childhood disorders.

Central to the phenotype of autism are patterns of focal and narrowed attention focus (3,4). Individuals with autism are often described as having a narrow focus of attention and interest, as well as acute perception for details. For example, unlike typically developing individuals, the cognitive processing style in autism appears to be biased toward local rather than global or configural information (3,5). This sometimes results in superior performance on tasks that benefit from these abilities (4). In brain imaging studies of adults with autism, increased activation of sensory ventral-occipitotemporal areas and decreased activation in prefrontal areas have been described as reflective of these processing differences in local versus global information (6).

A specific developmental account of the origins of the narrow processing style observed in autism relates to early emerging difficulties in visual attention (7). According to this view, the infant’s inability to flexibly switch the locus of attention leads to problems in self-regulation, as well as a decrease in orienting to socially relevant stimuli. Reduced orienting to social stimuli has been documented in preschool age children with autism (8,9). Moreover, in research on typical infants, individual differences in attention skills relate to differences in local versus global processing styles, and this association may have implications for the emergence of autism. As typically developing children begin to flexibly scan their environment and switch their attention between different objects, global forms become processed more rapidly and efficiently. Individual infants who exhibit a pattern of prolonged look durations on a single feature or object tend to rely more on local elements when processing visual stimuli (10,11).

Supporting evidence for the proposal that the development of visual attention plays a critical role in autism comes from studies using the gap-overlap task with children and adults. This task measures flexibility in attention switching in response to the presentation of a peripheral target in the presence or absence of a central fixation stimulus. A disengagement index is typically derived from this task measuring the cost of disengaging from a central stimulus to orient to a peripheral one. Thus, the gap task is also thought to reflect top-down control of attention (12). A number of studies using this task have demonstrated impairments in children and adults with autism both behaviorally as well as neurophysiologically (13,14).

Little is known about the early development of visual attention in autism because the condition is rarely diagnosed before 2 years of age. Converging lines of evidence indicate that general deficits, as well as specific precursors to some symptoms, are present early on in autism [reviewed in (15)]. By the age of diagnosis, several co-occurring impairments are clear and encompass both social and nonsocial domains. After the onset of these symptoms in the early years, different sets of abilities show varying trajectories of development. Some abilities, such as face processing, begin as seriously impaired, but over time, compensatory strategies and atypical neural systems may restore behavioral performance to within the typical range (7). Other deficits, such as executive dysfunction, may not be evident at younger ages but become clearer over development (7). In all cases, apart from aspects of behavior that define the disorder itself, substantial variability is seen in the resulting phenotype.

Recent studies focusing on infants at risk for autism by virtue of having an older diagnosed sibling have highlighted a range of atypical brain and behavioral functions not only in those infants who receive a diagnosis but also in the at-risk group as a whole (15). Atypical visual orienting has been reported by a number of studies. In our previous studies with a group of infants at risk at 9 to 10 months of age, we reported that relative to the control group, infants at risk were slower to disengage their fixation from a central stimulus to orient toward a peripheral distractor (16). In a second task, the same group required peripheral targets to remain on the screen for longer durations before infants reliably oriented to them (17).

Preliminary evidence from several longitudinal studies suggests that characteristics of visual attention are associated with autism symptoms emerging in toddlerhood. In one study, an increase, rather than the expected decrease, in latencies to disengage from a central fixation to a peripheral object between 6 and 12 months characterized those infants who went on to a provisional autism diagnosis at 2 years (18). Using a dimensional approach in quantifying the degree of emerging symptoms in the at-risk group as a whole, another study suggested that preference for a repetitive boring fixation stimulus was associated with more impairment in social and communication skills at 3 years of age (19).

In the current study, we tested the hypothesis that atypical visual disengagement during infancy is associated with autism-related outcomes in a new cohort of infants at risk for autism due to having an older sibling with the disorder [different from the group reported in our previously published work (16,17)]. Given the mixed evidence regarding the specificity of disengagement difficulties to autism, we also assessed if the same developmental pattern would be observed in infants who go on to exhibit some forms of developmental concerns without meeting criteria for a diagnosis.

## Methods and Materials

### Participants

One hundred four infants from the British Autism Study of Infant Siblings (www.basisnetwork.org) took part in the current study (54 at-risk, 21 male infants and 50 low-risk, 21 male infants). Along with several other measures, the infants were seen for the visual attention task when they were 6 to 10 months and again when they were 12 to 15 months. Subsequently, 52 (from 54) of those at risk for autism were seen for assessment around their second birthday and 53 were seen around their third birthday by an independent team. During the 36-month visit, a battery of clinical research measures was administered including the Autism Diagnostic Observation Schedule and the Autism Diagnostic Interview. Consensus ICD-10 criteria were used to ascertain diagnosis in a subgroup of infants at risk using all available information from all visits by experienced researchers (T.C., K.H., S.C., G.P.). Supplement 1 presents detailed participant characteristics including ascertainment of risk status, background measures at each visit, and outcome characterization including clinical classification. The at-risk groups were classified as having ASD (at-risk-ASD), other developmental concerns (at-risk-other), or typically developing (at-risk-typical). More specifically, infants were classified as at risk-other either because they did not meet criteria for a diagnostic classification for autism but scored above cutoff on the Autism Diagnostic Observation Schedule or Autism Diagnostic Interview or they had low IQ scores (<1.5 SD). Detailed characteristics of each group are presented in Supplement 1.

It is worth noting that the recurrence rate reported in the current study (32.1%) is higher than that reported in the large consortium paper recently published by Ozonoff *et al.* (20) (18.7%) and above the higher 95% confidence interval reported in that study (20). This is likely to reflect the modest size at-risk sample in the current study (*n* = 53). While recurrence rates approaching 30% have been found in other moderate size samples (21,22), these rates are sample specific and will likely not be generalizable, as findings from larger samples show autism recurrence rates converge between 10% and 20% (20,23). Similar procedures combining all information from standard diagnostic measures and clinical observation and arriving at a clinical best estimate ICD-10 diagnosis were used in the present study in line with other familial at-risk studies and were conducted by an experienced group of clinical researchers.

### Gap-Overlap Study at 6 to 10 Months and 12 to 15 Months

During their first and second visits, infants were administered a battery of tasks containing stimuli that varied across different tasks and with short breaks in between. The stimuli and procedure for the gap task were adapted from those reported in our previous study (16). Infants were presented with the stimuli on a 46-inch liquid crystal display monitor, while seated on their parent’s lap at 60 cm distance. Looking behavior was monitored and recorded through video from an adjacent room. All trials in this task began with a centrally presented animation. The animations, subtending around 13.8°×18°, expanded and contracted to attract the infant to the center before the onset of the trial. The peripheral target was presented randomly either to the right or the left of the central fixation stimulus at the eccentricity of 15°. Peripheral targets were always the same (a dynamic green balloon) subtending 6.3°×6.3°. The purpose of freezing the motion of the central stimulus during the overlap period was to better match the relative attractiveness of the two competing stimuli. Without this feature, the central and peripheral stimuli would have been unbalanced, leading to artificially lengthened disengagement values. The peripheral target remained displayed until the infant looked at it or until 2.5 seconds elapsed. Once the infant looked to the target or if the maximum duration was reached, an attractive animation of an animal with sound replaced the peripheral target and the next trial was presented. The rate of trial presentation was controlled by the experimenter.

In the baseline condition, the central fixation stimulus was extinguished and the peripheral target appeared simultaneously; in the overlap condition, the animated peripheral target appeared while the central fixation stimulus remained displayed (but not animated) so that the two stimuli overlapped. More overlap trials were presented because these trials are less likely to yield valid reaction times (infants may look away or become stuck on the central fixation), especially in atypical infants. The two conditions were presented pseudorandomly across two blocks that were identical except for the central fixation stimulus, to maintain the infant’s interest in the task. Trial presentation continued until the infant became fussy or until a maximum of 70 trials was reached. The session was repeated if infants were excessively fussy. Of the 104 infants, data from 4 (2 at-risk) were excluded at the 7-month visit and 6 (2 at-risk) at the 14-month visit due to loss to follow-up, technical problems, or excessive fussiness or fatigue. Table 1 presents the number of trials administered in the remaining group.

## Results

Video recordings of the infants’ looking behavior overlaid in real time with input from the stimulus screen were coded offline frame by frame by coders who established reliability of at least .9 (Cohen’s κ) for the validity of trials and correlation between saccadic reaction times was .87 on a training dataset. Coders were blind to outcome status of the infants but not to risk group membership. Trials were considered invalid if any of the following criteria were met: 1) the infant looked away from the screen at any point; 2) the infant did not look at the central stimulus immediately before the presentation of the peripheral stimulus; or 3) the infant blinked or looked away during the presentation of the peripheral stimulus. Saccadic reaction time data were analyzed for valid trials where the infant oriented toward the peripheral target after 100 to 1200 milliseconds of its appearance. If the infants did not look at the peripheral target within this period, reaction time was not analyzed and the trial was considered a failure to disengage (percentages for each group are presented in Table 1). The latter were considered to be an index of the likelihood of disengaging to orient to peripheral targets rather than the speed of orienting. Higher rates of disengagement failure would also reflect difficulties in flexibly switching attention.

**Figure 1:**
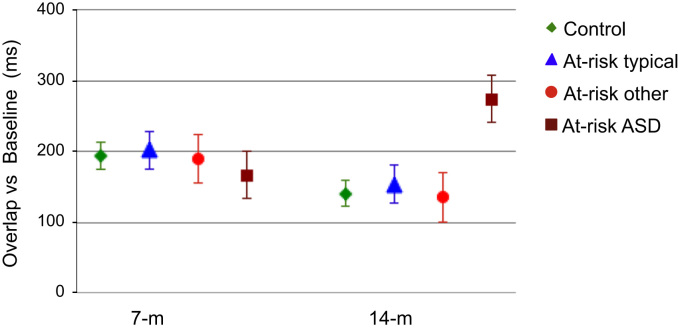
The 7-month (7-m) disengagement effect (overlap minus baseline) does not distinguish outcomes in toddlerhood, but a pattern of prolonged disengagement at 14 months (14-m) isolates the at-risk group who are subsequently diagnosed with autism. At-risk ASD, at-risk group classified as having autism spectrum disorder; At-risk other, at-risk group with other developmental concerns; At-risk typical, at-risk group typically developing.

Neither the risk groups (at-risk, control) nor groups defined based on 36-month outcomes (control, at-risk-ASD, at-risk-typical, at-risk-other) differed in the total number of trials completed or in the number of valid trials (all *p*s>.12) or in the percentage of trials where they failed to disengage (all *p*s>.15). Reaction times for each group in each condition are reported in Table 1. The disengagement effect, defined as the difference between the overlap and baseline conditions, was calculated for each group and is shown in Figure 1. Kolmogorov-Smirnov tests on the raw reaction times (RT) showed that the data within the two conditions were normally distributed (all *p*s>.11). While our focus was to test longitudinal developmental change between 7 and 14 months, in view of the age variability within each visit, we explored correlations between chronological age and RT measures in the two conditions within each visit. Age distribution within each visit was unrelated to RT (all *p*s>.1), consistent with previous studies supporting relatively stable reaction times across the ages studied (24–26).

To test our hypothesis, we used a generalized linear model (GLM) with the following repeated measures factors: condition (baseline, overlap), age (7 months, 14 months), and outcome group ascertained at 36 months (control, at-risk-typical, at-risk-other, at-risk-ASD) as a between-subjects factor. To test the specificity of any observed effects to the diagnostic outcomes, we included 36-month nonverbal T-score (NVT) (see Supplement 1 for details) as a continuous covariate in the GLM. Additional GLM assumptions were checked and found not to be violated. No significant interactions involving NVT were observed, but there was a main effect of NVT (*F*_1,86_ = 7.0, *p* = .01), indicating that Infants who had overall slower RTs exhibited poorer NVT scores at 36 months. There was no main effect of outcome group (*F*_3,86_ = .93, *p* = .43), indicating that overall reaction times did not differ among the groups.

A three-way interaction of condition×age×outcome was significant (Greenhouse-Geisser *F*_3,86_ = 4.2, *p* = .008). Planned comparisons focused on two potential explanations of the observed three-way interaction. First, we examined whether cross-sectional performance at 7 months or 14 months was associated with diagnostic outcomes (Figure 1). At 7 months and after controlling for NVT, there was a significant main effect of condition (*F*_1,86_ = 176.8, *p*<.01) but no main effect of outcome (*F*_3,86_ = 1.8, *p* = .16) or condition×outcome interaction (*F*_3,86_<1, *p* = .85). At 14 months and after controlling for NVT, there was a significant main effect of condition (*F*_1,86_ = 128.2, *p*<.01) and a significant condition×outcome interaction (*F*_3,86_ = 4.0, *p* = .01) but no main effect of outcome (*F*_3,86_ = 1.7, *p* = .18). Bonferroni corrected post hoc tests revealed that the groups did not differ from each other at 7 months in either condition. However, at 14 months, the at-risk-ASD group showed prolonged overlap RT relative to the control group (*p* = .001), the at-risk-typical group (*p* = .001), and the at-risk-other group (*p* = .04).

**Figure 2:**
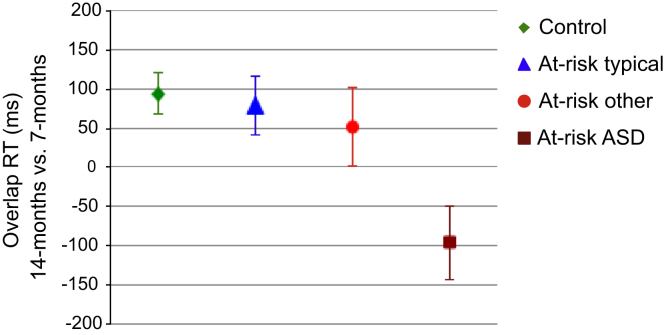
Estimated means in the overlap condition indicate that the control and at-risk typical groups show developmental gains in reaction time (RT), i.e., a smaller cost of disengagement at 14 months than at 7 months, whereas developmental gains are inconsistent in the at-risk group with later autism spectrum disorder (ASD) (and often negative), as well as in the other developmental concerns group. At-risk ASD, at-risk group classified as having autism spectrum disorder; At-risk other, at-risk group with other developmental concerns; At-risk typical, at-risk group typically developing.

The second planned comparison explored whether developmental changes between 7 months and 14 months within each group were associated with 36-month diagnostic outcomes, controlling for NVT. We ran separate GLM for each group with the repeated measures factors condition and age and NVT as a covariate. The main effect of age was significant in the control (*F*_1,41_ = 11.0, *p* = .002) and the at-risk-typical (*F*_1,19_ = 8.1, *p* = .01) groups but not in the at-risk-other (*F*_1,10_<1, *p* = .48) or at-risk-ASD (*F*_1,13_<1, *p* = .99) groups. Moreover, specific gains in the overlap versus baseline conditions were evident from a significant condition×age interaction in the control group (*F*_1,41_ = 5.3, *p* = .03) and the at-risk-typical (*F*_1,19_ = 6.1, *p* = .02) groups but not in the at-risk-other (*F*_1,10_<1, *p* = .37) or at-risk-ASD (*F*_1,13_ = 1.6, *p* = .22) groups. The developmental changes were most pronounced in the group later diagnosed with ASD where 40% of infants had longer disengagement latencies at 14 months relative to 7 months. Figure 2 shows the overall developmental gains across both conditions.

## Discussion

The majority of research on autism and its neural basis has been conducted on adults and children well after the full spectrum of symptoms has emerged. Indeed, little is known about the underlying processes through which this complex phenotype emerges. In view of this, interest has turned to the study of infant siblings of children with autism, in an attempt to discover early markers of the condition (7,20). Current evidence indicates that infants who receive a diagnosis of autism as toddlers show few differences in their behavior at 6 months but begin to show observable differences by about 12 months of age, characterized by the presence of atypical social and nonsocial behaviors documented by observational as well as experimental studies, including unusual eye contact, lack of orientation to name, and poor motor control (22,27). Our results support the conclusion of these previous findings by indicating that reduced flexibility in the control of visual attention is among the first early emerging features of autism, evident by at least 14 months of age.

Thus far, only direct measures of brain function have revealed reliable differences during the first year of infancy in the subgroup that were diagnosed with ASD in toddlerhood (28). This suggests that subtle characteristics of the condition emerge over the first year and gradually transform into the full-fledged condition during later development. The current results confirm that despite the lack of group differences when assessed within the first year, distinct developmental profiles indexing the control of visual attention characterize subgroups of infants at risk. Similar to the control group, at-risk infants who went on to typical outcomes at 36 months showed a consistent reduction in their reaction times, particularly in the overlap condition, suggesting developmentally appropriate improvements in the ability to flexibly switch visual attention. Toddlers with developmental concerns (but not an autism diagnosis) exhibited similar, albeit smaller and inconsistent, gains in the reaction time cost of disengagement between 7 and 14 months. While these age trends were nonspecific to outcome, those at-risk infants later diagnosed with ASD were not only inconsistent in their reaction time gains, but a significant proportion showed an increasing reaction time cost of disengagement over this period of development. These distinct profiles of reaction time differences were observable, despite the groups being comparable across several variables, including the number of trials produced and the likelihood of disengaging to orient to peripheral targets.

Although autism is characterized primarily by impairments in social skills and communication, the condition encompasses a complex phenotype. Frequently debated is the question of specificity of visual attention deficits to autism. Our findings suggest that at least within a group with familial risk for autism, atypical development of control of visual attention is indeed observed in those with ASD, as well as some of those with other developmental concerns. Within early infancy, distinct developmental trajectories characterize each of these groups. Our findings extend previous studies with smaller participant samples (16,18). Nevertheless, the current findings require replication in independent samples, especially in view of the high rate of ASD diagnosis in our sample relative to other samples (20). Even larger samples will help ascertain if these differences in visual attention are associated with specific features or subtypes of the conditions.

While causal links between looking behavior in infancy and later developmental outcome are tenuous, developmental models concerned with complex interactions among multiple developing systems may offer some reasons for the patterns observed in our study. Early emerging differences in cortical systems mediating visual orienting will modify the infant’s ability to select relevant information from the social and nonsocial environment. Such differences in earlier developing brain systems will have consequences for later developing ones, such as those mediating social cognition. Consequently, atypical modulation of early visual processing areas by top-down feedback would have far-reaching implications in modifying other perceptual, cognitive, and social processes that rely on these top-down systems (26).

Such links between visual attention and later social outcomes have previously been suggested. Goal-directed selection of information from the environment can be used to self-regulate arousal and affect (27). Beyond gathering information from others, active visual exploration provides information for the infant about the integration of self-attention and other attention. As such, the current results raise important questions regarding the origins of the neurobiological processes leading to either a plateau or losses in the development of visual orienting beginning within the first year and clearly evident within the second year.

One possibility for clarifying interactions between social- and attention-developing brain systems is to manipulate characteristics of either the central fixation stimulus or the targets. While our study did not address this possibility, previous research with young children with autism suggests that such manipulations modulate visual attention performance (29,30). For example, children with autism are faster at disengaging from face relative to nonface central stimuli, a pattern taken to suggest reduced engagement with faces (30). Whether this attention modulation of social response begins earlier in life needs to be considered in future studies.

*The research is supported by The United Kingdom Medical Research Council (G0701484) and the British Autism Study of Infant Siblings (BASIS) funding consortium led by Autistica (www.basisnetwork.org) to MHJ. Further support for some of the authors is from Autism Speaks and European Cooperation in Science and Technology ( COST) action BM1004. The BASIS team in alphabetical order: Simon Baron-Cohen, Rachel Bedford, Patrick Bolton, Susie Chandler, Holly Garwood, Teodora Gliga, Kristelle Hudry, Greg Pasco, Andrew Pickles, Leslie Tucker, and Agnes Volein. We are very grateful for the enormous contributions BASIS families have made toward this study*.

*The authors report no biomedical financial interests or potential conflicts of interest*.

## Figures and Tables

**Table 1:** Number of Trials and Mean Reaction Times in the Group Retained for Analysis

						7 Months						14 Months				
		*n*	Total Trials		Valid Trials Baseline	Valid Trials Overlap	% No Disengagement	RT Baseline	RT Overlap	*n*	Total Trials	% No Disengagement	Valid Trials Baseline	Valid Trials Overlap	RT Baseline	RT Overlap
Control	Mean	48	47.3		12.7	14.9	.17	467.4	641.9	46	47.4	.15	12.1	15.2	427.3	565.5
	SD		2.4		2.3	6.0	.11	92.7	139.4		2.4	.08	2.8	5.2	92.4	107.0
At-Risk Combined	Mean	52	47.1		12.7	15.5	.15	432.8	623.8	52	49.0	.16	12.3	12.8	427.9	607.4
	SD		3.6		2.4	5.1	.09	61.5	128.4		8.3	.11	3.1	5.8	81.4	165.9
At-risk-typical	Mean	23	47.0		12.4	15.2	.15	428.5	610.7	22	46.5	.14	11.5	13.4	418.7	556.0
	SD		4.5		2.6	5.2	.09	63.2	141.7		3.4	.11	3.3	5.9	85.6	137.3
At-risk-other	Mean	12	48.0		13.0	17.2	.11	436.0	628.2	12	50.5	.16	14.1	13.8	440.7	577.1
	SD		.7		2.0	5.5	.09	67.0	144.4		9.6	.11	2.8	6.0	76.5	148.8
At-risk-ASD	Mean	16	47.1		13.1	14.9	.17	435.4	629.1	17	51.1	.18	12.7	11.7	428.3	697.1
	SD		2.4		2.3	4.7	.11	60.3	95.9		11.1	.12	2.1	5.9	84.5	186.8

ASD, autism spectrum disorder; RT, reaction time.

## Footnotes

Appendix A Supplementary material cited in this article is available online.
