# Supplementary material for: Disengagement of Visual Attention in Infancy is Associated with Emerging Autism in Toddlerhood
Source: Biol Psychiatry. 2013 Aug 1;74(3):189–94. doi: 10.1016/j.biopsych.2012.11.030 (PMC3715700; doi:10.1016/j.biopsych.2012.11.030)
Supplement: Supplementary file 1 — Supplementary Material [file mmc1.pdf]

Disengagement of Visual Attention in Infancy is Associated with Emerging Autism in Toddlerhood

Supplemental Information

Participants and Clinical Characterization

Recruitment, ethical approval (NHS NRES London REC 08/H0718/76) and informed consent, as well as background data on participating families, were made available for the current study through The British Autism Study of Infant Siblings (BASIS), a UK collaborative network facilitating research with infants at-risk for autism (www.basisnetwork.org). Families enroll from various regions when their babies are younger than 5 months of age and they are invited to attend multiple research visits until their children reach three years of age or beyond. Each visit lasts a day or two and is adapted to meet the families' needs. Measures collected are anonymized and shared among scientists to maximize collaborative value and to minimize burden on the families. A clinical advisory team of senior consultants works closely together with the research team/s and, if necessary, with the family's local health services, to ensure that any concerns about the child arising during the study are adequately addressed.

One hundred and four infants from BASIS took part in the current study (54 at-risk, and 50 low-risk). Twenty-one of the at-risk infants were male, 33 were female. Twenty-one of the low-risk infants were male, 29 were female. Along with several other measures, the infants were seen for the task at the Centre for Brain and Cognitive Development when they were 6 to 10-months of age and again at 12-15 months. Subsequently, 52 (from 54) of those at-risk for autism spectrum disorder (ASD) were seen for assessment around their second birthday (mean = 23.9 months, SD = 1.2) and 53 around their third birthday (mean = 37.7 months, SD = 3.0), by an independent team at the Centre for Research in Autism and Education, Institute of Education.

Confirmation of Risk Status in the Older Sibling

At the time of enrollment, none of the infants had been diagnosed with any medical or developmental condition. Infants at-risk all had an older sibling (hereafter, proband) with a community clinical diagnosis of ASD (or in 4 cases, a half-sibling), and in 3 cases 2 probands with an ASD. Forty-five probands were male, 9 were female. Proband diagnosis was confirmed by two expert clinicians (PB, TC) based on information using the Development and Wellbeing Assessment (DAWBA) (1) and the parent-report Social Communication

Questionnaire (SCQ) (2). Most probands met criteria for ASD on both the DAWBA and SCQ (n = 44). While a small number scored below threshold on the SCQ (n = 4), no exclusions were made, due to meeting threshold on the DAWBA and expert opinion. For 2 probands, data were only available for either the DAWBA (n = 1) or the SCQ (n = 1). For 4 probands, neither measure was available (aside from parent-confirmed local clinical ASD diagnosis at intake). Parent-reported family medical histories were examined for significant medical conditions in the proband or extended family members, with no exclusions made on this basis.

Infants in the low-risk group were recruited from a volunteer database at the Birkbeck Centre for Brain and Cognitive Development. Inclusion criteria included full-term birth (with one exception), normal birth weight, and lack of any ASD within first-degree family members (as confirmed through parent interview regarding family medical history). All low-risk infants had at least one older-sibling (in 3 cases, only half-sibling/s). Twenty-eight of the older siblings were male, 22 were female. Screening for possible ASD in these older siblings was undertaken using the SCQ, with no child scoring above instrument cut-off for ASD ($\geq$15) (one score was missing).

Background Characterization Measures

Two measures of general developmental level were obtained for the infants and toddlers at each visit. The Mullen Scales of Early Learning (MSEL) (3) is a direct assessment of verbal and non-verbal abilities appropriate for children from birth to 6 years. Scores across four domains – Visual Reception, Fine Motor, Receptive Language, and Expressive Language – are combined to yield an overall Early Learning Composite (ELC; mean = 100, SD = 15). Gross motor skills are also assessed but do not contribute to the ELC. An estimate of non-verbal developmental ability was computed by averaging the T scores (mean = 50, SD = 10) for Visual Reception and Fine Motor subscales. The Vineland Adaptive Behavior Scales (VABS) (4) is a parent-report measure of everyday skills in the domains of Communication, Daily Living Skills, Social Interaction, and Motor Skills. These combine to yield an Adaptive Behavior Composite (mean = 100, SD = 15).

These developmental assessments were undertaken at each of the visits, when infants were 6- to 10-months, 12 to 15-months, and again around the second and third birthday, each time by independent research teams. While the MSEL is always administered directly with the child, the VABS has alternative administration formats. The Parent/Caregiver Rating Form (i.e., questionnaire booklet) was used at the 6- to 10-month and 12- to 15-month visits,

and the Survey Interview Form was used at the 24-month and 36-month visits. Scores from these measures are presented in Table S1.

Outcome Characterization of the At-Risk and Low-Risk Groups

Alongside the standard measures of cognitive (MSEL) and adaptive (VABS) development taken at each visit, at 24 months (at-risk group only; 50 Module 1, 2 Module 2) and 36 months (both groups; Table S1, 3 Module 1, 98 Module 2) a semi-structured play assessment, the Autism Diagnostic Observation Schedule (ADOS) (5) was used to assess autism-related social and communication behavioral characteristics. This was augmented at 36 months (at-risk group only) with the parent-report Autism Diagnostic Interview (ADI) (6).

Characterization of outcomes in the at-risk cohort at 36-months was done by ascertaining three sub-groups (Table S1): Those who were typically-developing, those classified as having ASD, and those exhibiting some form of developmental concerns. For the at-risk group consensus ICD-10 (7), ASD (including childhood autism; atypical autism, other pervasive developmental disorder (PDD)) was diagnosed using all available information from all visits by experienced researchers (TC, KH, SC, GP), hereafter ‘At-risk-ASD’. From the 53 toddlers assessed at 36-months, 17 (11 boys, 6 girls) met criteria for an ASD diagnosis (32.1%). Given the young age of the children, and in line with the proposed changes to DSM-5 (8), no attempt was made to assign specific sub-categories of PDD/ASD diagnosis. Another subgroup of toddlers from the at-risk group who were classified as not having ASD were considered to still have other developmental concerns (‘At-risk Other’). These were 12 toddlers (22.6%; 3 boys, 9 girls) who either scored above the ADOS or ADI (9) cut-off for ASD or scored <1.5 SD on the Mullen ELC or Receptive Language and Expressive Language subscales but did not meet ICD-10 criteria for an ASD (9 scored > ADOS cut-off, 1 > ADOS cut-off and <1.5 SD Mullen ELC cut-off, 1 > ADI cut-off, and 1 <1.5 SD Mullen ELC cut-off). The remaining 24 (45.4%; 7 boys, 17 girls) at-risk children meet neither of the above criteria and were considered to be clearly typically developing (‘At-risk Typical’).

It is worth noting that the toddlers with ASD are mostly relatively high functioning. This pattern is an emerging finding from several sibling studies (10, 11) and likely reflects differences between ASD children ascertained from a familial at-risk design and clinically referred cohorts, who often include a considerably greater proportion of children with ASD with an intellectual disability, and the generalizability of the current findings to such samples needs to be demonstrated.

Table S1. Participant characteristics

		Control	At-Risk			
			Combined	At-Risk no ASD	At-risk ASD	No ASD-Other concerns
**Visit**	**Measure**	**Mean (SD)** ***n***	**Mean (SD)** ***n***	**Mean (SD)** ***n***	**Mean (SD)** ***n***	**Mean (SD)** ***n***
6-10 months	Age at visit (months)	7.4 (1.2) 50	7.3 (1.2) 54	7.1 (1.2) 24	7.5 (1.2) 17	7.3 (1.1) 12
	Mullen ELC SS	104.4 (11.3) 50	94.0 (12.8) 53	96.1 (11.8) 24	92.1 (17.3) 16	92.8 (8.1) 12
	Mullen NV T-score	56.2 (7.1) 50	51.5 (8.4) 53	52.6 (8.6) 24	49.9 (9.8) 16	51.3 (6.3) 12
	VABS ABC SS	101.8 (13.7) 49	92.1 (14.8) 53	95.7 (17.8) 23	90.0 (13.4) 17	87.6 (9.0) 12
12-15 months	Age at visit (months)	13.9 (1.3) 48	13.7 (1.6) 53	13.5 (1.7) 23	13.9 (1.6) 17	13.5 (1.2) 12
	Mullen ELC SS	106.1 (15.7) 47	97.4 (17.9) 53	103.3 (18.1) 23	89.2 (18.3) 17	99.8 (11.3) 12
	Mullen NV T-score	58.4 (8.3) 47	53.1 (10.3) 53	54.5 (10.7) 23	49.4 (10.9) 17	56.7 (6.3) 12
	VABS ABC SS	100.8 (8.9) 45	91.5 (13.8) 51	95.6 (10.3) 21	87.5 (13.7) 17	90.6 (18.5) 12
24 months	Age at visit (months)	23.9 (0.7) 47	23.9 (1.2) 52	23.9 (1.3) 24	24.0 (1.0) 16	23.8 (1.1) 12
	Mullen ELC SS	116.0 (14.0) 42	102.3 (19.8) 52	105.4 (17.5) 24	97.8 (24.7) 16	102.0 (16.8) 12
	Mullen NV T-score	56.9 (8.8) 43	51.6 (9.7) 52	53.7 (7.9) 24	49.4 (11.3) 16	50.2 (10.5) 12
	VABS ABC SS	108.2 (12.0) 47	101.5 (10.6) 52	103.5 (9.9) 24	100.0 (12.8) 16	99.3 (8.8) 12
	ADOS Communication	--	2.1 (1.6) 52	1.3 (1.2) 24	3.2 (1.8) 16	2.3 (1.3) 12
	ADOS Social	--	4.3 (3.0) 52	3.0 (2.8) 24	6.6 (2.9) 16	3.8 (1.6) 12
	ADOS Total	--	6.4 (4.3) 52	4.4 (3.8) 24	9.8 (4.3) 16	6.0 (2.1) 12
36 months	Age at visit (months)	38.2 (3.1) 48	37.7 (3.0) 53	38.1 (3.9) 24	37.8 (2.1) 17	36.7 (1.8) 12
	Mullen ELC SS	115.8 (16.3) 48	105.4 (21.5) 52	113.5 (13.3) 24	94.8 (28.5) 16	103.4 (19.0) 12
	Mullen NV T-score	57.8 (9.9) 48	52.6 (13.0) 52	57.1 (9.3) 24	45.3(15.8) 16	53.2 (12.1) 12
	VABS ABC SS	106.4 (9.1) 48	96.4 (12.2) 53	101.3 (8.7) 24	90.1 (14.6) 17	95.7 (10.8) 12
	ADOS Communication	2.5 (1.5) 48	3.3 (2.2) 53	2.0 (1.2) 24	4.2 (2.5) 17	4.8 (1.9) 12
	ADOS Social	3.2 (3.1) 48	4.9 (3.5) 53	2.0 (1.5) 24	7.4 (2.7) 17	7.3 (2.6) 12
	ADOS Total	5.6 (4.3) 48	8.3 (5.3) 53	4.0 (2.2) 24	11.7 (4.7) 17	12.1 (4.1) 12
	% above ADOS ASD threshold	22.9% 48	43.4% 53	1.6 (1.7) 24	76.5% 17	83.4% 12
	ADI Social	--	4.5 (5.3) 52	2.2 (1.8) 24	9.8 (5.5) 16	3.4 (4.9) 12

		**Control**	**At-Risk**			
			**Combined**	**At-Risk no ASD**	**At-risk ASD**	**No ASD-Other concerns**
**Visit**	**Measure**	**Mean (SD) *n***	**Mean (SD) *n***	**Mean (SD) *n***	**Mean (SD) *n***	**Mean (SD) *n***
	ADI Communication	--	4.4 (4.8) 52	0.5 (0.9) 24	8.4 (5.1) 16	3.6 (5.5) 12
	ADI Beh/Rep Int	--	1.6 (2.0) 52	0.69 (1.1) 36	3.6 (2.2) 16	1.1 (1.3) 12
	% above ADI ASD threshold	--	17.3% 52	0% 24	50.0% 16	8.3% 12

ABC, Adaptive Behavior Composite; ADI, Autism Diagnostic Interview; ADOS, Autism Diagnostic Observation Schedule; ASD, autism spectrum disorder; Beh/Rep Int, Restricted, Repetitive and Stereotyped Patterns of Interest; ELC, Early Learning Composite; NV, non-verbal; SS, standard score; VABS, Vineland Adaptive Behavior Scales.

Supplemental References

1. Goodman R, Ford T, Simmons H, Gatward R, Meltzer H (2000) Using the Strengths and Difficulties Questionnaire (SDQ) to screen for child psychiatric disorders in a community sample. *Br J Psychiatry* 177:534-539.
2. Rutter M, Bailey A, Lord C (2003): *Social Communication Questionnaire.* Los Angeles, CA: Western Psychological Services.
3. Mullen EM (1995) *Mullen Scales of Early Learning*. Circle Pines, MN: American Guidance Service.
4. Sparrow SS, Cicchetti VD, Balla AD (2005) *Vineland II: A Revision of the Vineland Adaptive Behavior Scales: Survey/Caregiver Form.* Circle Pines, MN: American Guidance Service.
5. Lord C, Risi S, Lambrecht L, Cook EH Jr, Leventhal BL, DiLavore PC, *et al.* (2000): The Autism Diagnostic Observation Schedule-Generic: A standard measure of social and communication deficits associated with the spectrum of autism. *J Autism Dev Disord* 30:205-23.
6. Lord C, Rutter M, Le Couteur A (1994): Autism Diagnostic Interview-Revised: a revised version of a diagnostic interview for caregivers of individuals with possible pervasive development disorders. *J Autism Dev Disord* 24:659-85.
7. World Health Organisation (1993): *Mental Disorders: A Glossary and Guide to their Classification in Accordance with the 10th Revision of the International Classification of Diseases: Research Diagnostic Criteria (ICD-10).* Geneva: WHO.
8. American Psychiatric Association (2012): *Diagnostic and Statistical Manual of Mental Disorders, 5th edition.* Washington, DC: American Psychiatric Press. Available at: www.dsm5.org.
9. Risi S, Lord C, Gotham K, Corsello C, Chrysler C, Szatmari P, *et al.* (2006): Combining information from multiple sources in the diagnosis of autism spectrum disorders. *J Am Acad Child Adolesc Psychiatry* 45:1094-1103.
10. Paul R, Fuerst Y, Ramsay G, Chawarska K, Klin A (2011): Out of the mouths of babes: vocal production in infant siblings of children with ASD. *J Child Psychol Psychiatry* 52:588-98.
11. Yoder P, Stone WL, Walden T, Malesa E (2009): Predicting social impairment and ASD diagnosis in younger siblings of children with autism spectrum disorder. *J Autism Dev Disord* 39:1381-191.
